# The Malcolm Campbell Memorial Meeting

**Published:** 1975-04

**Authors:** 


### A Meeting of the South Western Ophthalmological Society

**was held on Friday, 8th June, 1973**

### in memory of Dr. A. M. G. Campbell

**1909 - 1972**

**at the Main Lecture Theatre, Phase I, Bristol Royal Infirmary**

In addition to the five papers printed in this issue of the Journal, the following communications were given to the Society:—

| | |
|---|---|
| Professor Refsum | 'Refsum's syndrome' |
| Dr. Ian McDonald (London) | 'The use of the pattern evoked in the diagnosis of optic nerve disease' |
| Dr. R. Langton Hewer (Bristol) | 'Optic atrophy in friedrich's ataxia' |
| Mr. Michael Saunders (London) | 'Paralysis of horizontal gaze from pontine lesions' |
| Dr. L. Liversedge (Manchester) | 'Ocular myopathies' |

